# miR-34a regulates cell proliferation, morphology and function of newborn neurons resulting in improved behavioural outcomes

**DOI:** 10.1038/cddis.2014.589

**Published:** 2015-01-29

**Authors:** C Mollinari, M Racaniello, A Berry, M Pieri, M C de Stefano, A Cardinale, C Zona, F Cirulli, E Garaci, D Merlo

**Affiliations:** 1 Institute of Translational Pharmacology, National Research Council, Rome, Italy; 2 Department of Cell Biology and Neuroscience, Istituto Superiore di Sanità, Rome, Italy; 3 Department of Experimental Medicine and Surgery, University of Rome ‘Tor Vergata', Rome, Italy; 4 Department of System Medicine, University of Rome ‘Tor Vergata', Rome, Italy; 5 IRCCS San Raffaele Pisana, Rome, Italy

## Abstract

miR-34a is involved in the regulation of the fate of different cell types. However, the mechanism by which it controls the differentiation programme of neural cells remains largely unknown. Here, we investigated the role of miR-34a in neurogenesis and maturation of developing neurons and identified Doublecortin as a new miR-34a target. We found that the overexpression of miR-34a *in vitro* significantly increases precursor proliferation and influences morphology and function of developing neurons. Indeed, miR-34a overexpressing neurons showed a decreased expression of several synaptic proteins and receptor subunits, a decrement of NMDA-evoked current density and, interestingly, a more efficient response to synaptic stimulus. *In vivo*, miR-34a overexpression showed stage-specific effects. In neural progenitors, miR-34a overexpression promoted cell proliferation, in migratory neuroblasts reduced the migration and in differentiating newborn neurons modulated process outgrowth and complexity. Importantly, we found that rats overexpressing miR-34a in the brain have better learning abilities and reduced emotionality.

microRNAs (miRNAs) are small non-coding RNAs that regulate post-transcriptional expression of genes involved in a wide range of developmental and pathological processes.^1, 2^

miR-34s are an evolutionary conserved family with three members in vertebrate genome (miR-34a, miR-34b and miR-34c) and single orthologues in invertebrate species. miR-34a, initially identified as a potential tumour suppressor in neuroblastoma,^3^ is ubiquitously expressed with the highest expression in brain.^4, 5, 6^ In developing brain, it is detected in progenitor cell niches surrounding the lateral ventricle during cortical neurogenesis.^7^ In addition, miR-34a has been shown to negatively regulate *in vitro* dendritic branching of developing cortical neurons altering the expression of a number of synaptic proteins.^8, 9^ Further, miR-34a regulates mouse neural stem cell differentiation,^10^ and the expression of the synaptic plasticity-related gene Arc in rat hippocampal neurons.^11^ Thus, there is growing evidence that miR-34a is involved in mammalian neurogenesis, neuronal differentiation and synaptogenesis, although so far, relatively few miR34a/target interactions have been experimentally validated in the nervous system.

In the adult mammalian hippocampus, neurogenesis occurs throughout the lifetime providing a supply of newly generated neurons that undergo maturation processes, display enhanced plasticity and become synaptically integrate into the local circuitry.^12^ Importantly, neurogenesis has been correlated with learning and memory,^13^ and is dynamically regulated by physiological and pathological stimuli.^14^ Furthermore, alterations in adult neurogenesis are a common pathological feature in several human neurodegenerative diseases. For instance, in Alzheimer's disease, hippocampal neurogenesis has been suggested to increase,^15^ and several studies indicate that, during acute or chronic neurodegeneration, neurogenesis is increased and is tightly regulated to replace damaged neurons at the lesion site.^16^ Thus, it is a current goal to gain better understanding of factors and signalling mechanisms controlling adult neurogenesis and to translate such knowledge into designing new therapeutic strategies.

Here, we investigated the functional effects of miR-34a in rat brain and demonstrated that miR-34a is involved in the control of adult neurogenesis and maturation of developing neurons both *in vitro* and *in vivo*. Moreover, miR-34a overexpression is associated with a more efficient neuronal response to a synaptic stimulus, cognitive improvement and reduced emotionality. Furthermore, we identified Doublecortin (DCX) as a new miR-34a target whose downregulation may be responsible, at least in part, of miR-34a-mediated effects.

## Results

### rAAV-mediated miR-34a overexpression increases cortical precursor cell proliferation *in vitro*

First, we measured miR-34a expression levels at different days *in vitro* (DIV) in neuronal precursors isolated from cortex of E15 rat embryos, using real-time PCR. We found that the levels of the endogenous miR-34a dramatically increase in the initial stages of development then remaining high and stable in the tardive stages of differentiation and maturation (Supplementary Figure S1a).

After having established that dynamic changes in the expression of miRNA-34a occur during precursor cell differentiation, we investigated whether overexpression of miR-34a levels could affect neuronal formation and maturation. To this aim, we exploited a recombinant adeno-associated (rAAV)-mediated gene delivery system to overexpress miR-34a gene (pri-miR-34a) along with the EGFP, under two independent constitutive promoters and, as a control, an AAV empty vector overexpressing only EGFP (Supplementary Figure S1b). rAAV infection was performed on purified cortical precursors soon after plating and vessel attachment (DIV 0). We first verified, by real-time PCR, that miR-34a was overexpressed after rAAV infection (Supplementary Figure S1c). We found that miR-34a was dramatically upregulated, on average 10-fold, after infection and confirmed its co-expression with the marker EGFP. Similarly, EGFP overexpression was high in cultures infected with the empty vector (Supplementary Figure S1c). The elevated levels of expression of miR-34a led us to investigate whether miR-34a could be found in exosomal preparations of overexpressing cultures. Indeed, we found a strong increase of miR-34a exosomal preparation, as compared with uninfected cultures (Supplementary Figure S2), suggesting that its regulatory effects could be also mediated through a cell non-autonomous mechanism.^17^

**Figure 1:**
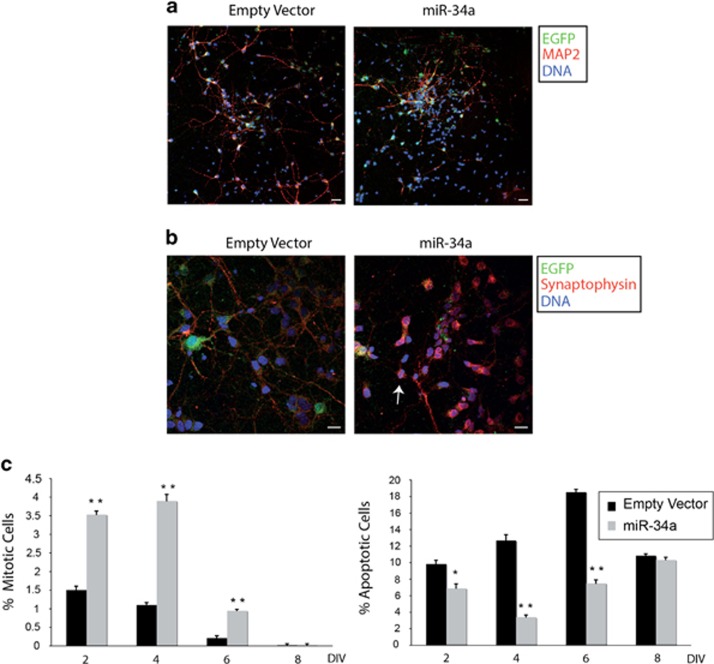
miR-34a overexpression increases the number of mitotic precursors in the early days of culture. (**a**) Low-power magnification of triple-labelled confocal images from cultures transduced with the empty vector or the miR-34a vector. Cells positive for EGFP (green) and MAP2 (red) are shown. In miR-34a transduced cells, it is possible to note a higher cellular density, as indicated by the nuclei stained with DAPI (blue). Note that, although the fluorescence of the EGFP is low in cells at DIV 4, it indicates that viral infection has properly occurred. Scale bar: 20 *μ*m. (**b**) Representative confocal images (DIV 3) positive for EGFP (green), immunolabelled for synaptophysin (red) and counterstained with DNA (blue). In miR-34a overexpressing cultures, a mitotic cell in metaphase (white arrow) is evident among the differentiating cells. Scale bar: 10 *μ*m. (**c**) Quantification of cells in mitosis (left) and in apoptosis (right) of transduced cultures at different DIV. In the early DIV, miR-34a transduced cultures show a significant increase in proliferating precursor cells, suggesting a delay in the onset of differentiation. The results are expressed as a percentage of cell detected, divided by the total number of cells in each field. Bars represent means±S.E.M. **P<0.01; *P<0.05 compared with control values

**Figure 2:**
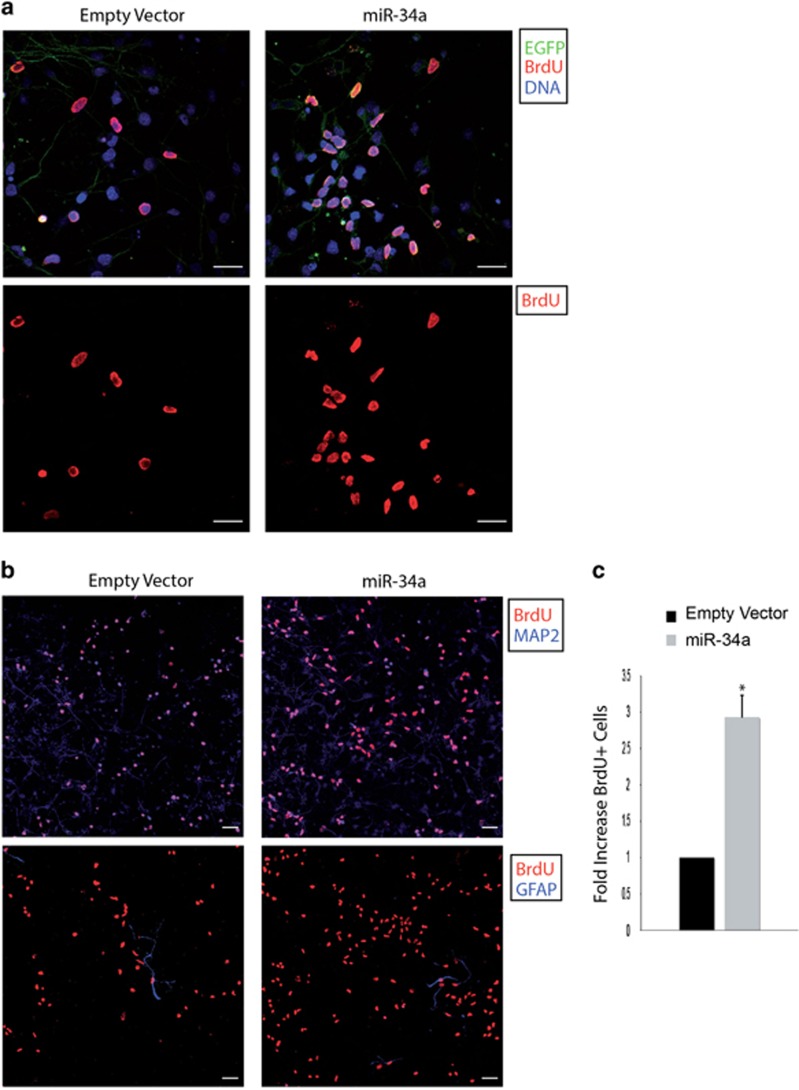
miR-34a induces precursor proliferation *in vitro* after BrdU labelling. (**a**) Higher-magnification confocal images positive for EGFP (green), immunolabelled with anti-BrdU (red) and counterstained with DAPI (blue). Merged images (top panel) and the corresponding single red channel images (lower panel) are shown. In miR-34a overexpressing cultures, more cells incorporated BrdU indicating a higher proliferative capacity. Scale bar: 10 *μ*m. (**b**) Low-power magnification of double labelled confocal images from cultures transduced with the empty vector or the miR-34a vector. Transduced cells (DIV 4) positive for anti-BrdU (red) were immunostained for MAP2 (blue) (upper panel) or GFAP (blue) (lower panel). The immunolabelling for MAP2 confirms that cells positive for BrdU are mainly neurons and these cells are present in a higher number in miR-34a overexpressing cultures as compared with the control cultures. Few cells incorporating BrdU result to be glia, as shown by the staining with the anti-GFAP antibody. Scale bar: 10 *μ*m. (**c**) Quantitative data are expressed as fold increase in BrdU+ cells. miR-34 overexpression results in a marked increase in the number of BrdU+ cells. The results are expressed as fold change with respect to control cultures (empty vector). Bars represent means±S.E.M. *P<0.01 compared with control values

Because miR-34a has a well-demonstrated effect on cell proliferation,^18^ we analysed whether miR-34a overexpression could influence the proliferative state also of neuronal precursors. It is noteworthy that miR-34a overexpressing cultures, examined under a fluorescence microscope, showed a cell confluence higher as compared with control cultures infected with the empty vector, suggesting an increase in the total number of cells (Figure 1a). Indeed, we observed that, from the first days of miR-34a overexpression, cortical cultures had a higher number of dividing precursors along with a lower number of apoptotic cells (Figures 1b and c). Therefore, we used BrdU labelling to establish the proliferative effect of miR-34a in neuronal precursors. First, BrdU was added the day after cell infection (DIV 1), then cultures were stained with an anti-BrdU antibody at different DIV. As illustrated in Figure 2a, miR-34a infected cultures show a greater BrdU incorporation as compared with empty vector infected cells. Next, we performed double-labelling experiments using the neuronal marker MAP2 and the glial marker GFAP.^19^ As shown in Figures 2b and c, upregulation of miR-34a induces an increased BrdU incorporation (2.8-fold) in neurons (Figure 2b, upper panel) with very few BrdU+ glial cells (Figure 2b, lower panel), thus demonstrating that miR-34a acts as a mitogen for neuronal committed precursors.

### miR-34a regulates neuronal morphology

**Figure 3:**
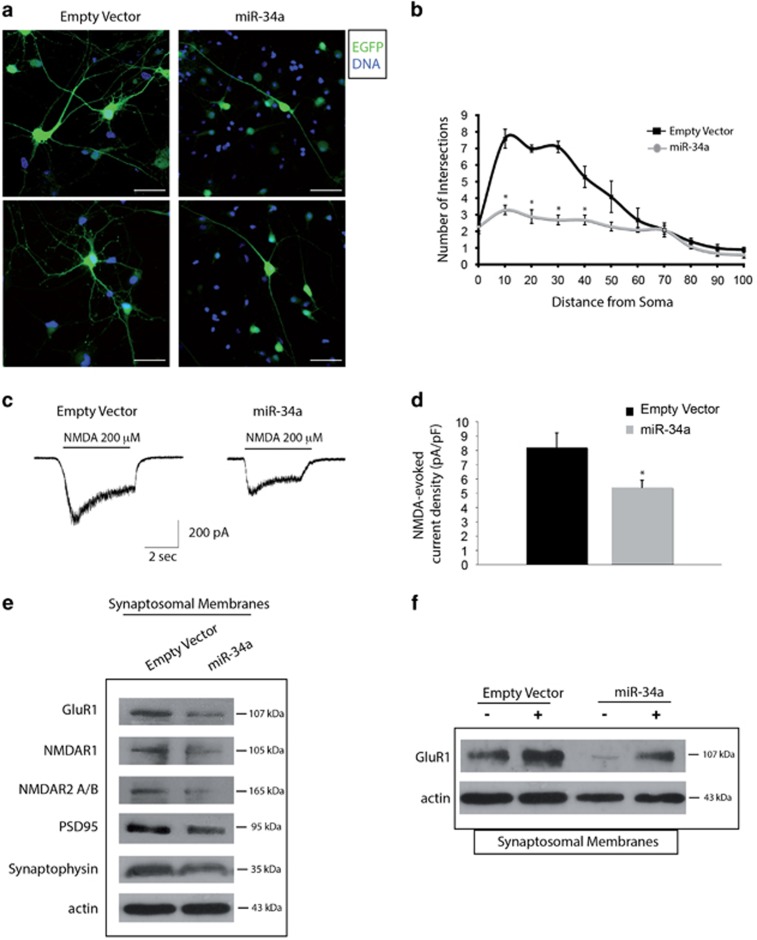
miR-34a affects neurite outgrowth and induces electrophysiological and biochemical changes in maturing cortical neurons. (**a**) Representative confocal images of developing cortical neurons (DIV 7) positive for the EGFP (green) and stained with DAPI for nuclei detection (blue). Empty vector neurons show a higher number of neurites emanating from the cell body compared with the simpler neuritic morphology of miR-34a-infected neurons. Scale bar: 10 *μ*m. (**b**) The Sholl analysis performed on neurons (DIV 9) confirms a lower neurite complexity as indicated by a significant reduction in the number of intersections in the range of 10–40 *μ*m distance from cell body. *P<0.05 compared with control values. (**c**) Representative whole-cell response to NMDA (200 *μ*M) recorded at −60 mV in the presence of glycine (3 *μ*m) in the two cell types. Bars indicates duration of NMDA application. (**d**) Histogram of data on peak current amplitude normalized to cell capacitance from the empty vector and in mir-34a cells. The response of cells with the empty vector was significantly greater than cells with miR-34a (*P*<0.05, Student's *t* test). The data were derived from four cell preparations (empty vector cells *n*=28; mir-34a cells *n*=25). (**e**) Western blot analysis on synaptosomal membranes purified from infected cultures confirms a reduction of PSD95, synaptophysin along with NMDA receptor subunit GluR1, NMDAR1 and NMDAR2A/B, in miR-34a cultures as compared with controls. *β*-Actin was used as a loading control. (**f**) Western blot analysis on synaptosomal membranes purified from infected neurons unstimulated or after chemical LTP induced by forskolin/rolipram stimulation. Although synaptosomal membranes from miR-34a infected neurons have a lower threshold of expression of GluR1 receptor subunit, after chemical stimulation, neurons are capable of recruiting NMDA ion channels for LTP even more efficiently than controls. *β*-Actin was used as a loading control

Next, we focused on the morphology of infected developing neurons and analysed the complexity of neurites. Indeed, we found that miR-34a overexpressing neurons are morphologically different from neurons infected with the empty vector. Interestingly, as shown in Figure 3a, empty vector-infected neurons have more prominent neurites emanating from the cell body, thus acquiring a multipolar aspect, whereas miR-34a overexepressing neurons show a significant reduction in neurite complexity as confirmed by Sholl analysis (Figure 3b).^20^ On the contrary, no significant differences were found in the total length of major neurites (neurons DIV 7, 115.3±21.8 *μ*m for miR-34a neurons; 109.4±14.3 *μ*m for empty vector neurons). Together, these results indicate that miR-34a has a role in controlling neurite complexity.

### miR-34a overexpressing neurons show modified physiological properties

We then investigated whether the effects of miR-34a overexpression on neurite morphology had functional consequences, in particular at the electrophysiological level. Therefore, we analysed the current in response to NMDA application (0.2 mM) in the presence of glycine (3 mM) in control and miR-34a cells by whole-cell patch-clamp recordings at a constant voltage of −60 mV. Bath application of NMDA for 2–10 s induced inward currents in all tested neurons, voltage clamped at −60 mV. In addition, the responses of both cell types were blocked to a similar extent by the NMDA antagonist D-2-phosphonopentanoic acid and at the negative potentials by Mg^2+^, which blocks the NMDA channel (data not shown). Interestingly, the peak amplitude of the NMDA current in miR-34a cells was greatly suppressed as compared with control neurons (Figures 3c and d) by normalizing NMDA-induced current to cell capacitance (empty vector cells, 8.2±5.3 pA/pF, *n*=28; miR-34 A cells, 5.4±2.5 pA/pF *n*=25; *P*<0.05; all cells were recorded at 11 DIV).

Western blot analysis on synaptosomal membranes from infected cortical cultures showed that miR-34a upregulation causes a lower expression of several receptor subunits including GluR1 (46% reduction), NMDAR1 (34%), NMDAR2A/B (55%) and several synaptic proteins such as PSD95 (38%), synaptophysin (33%) and synaptotagmin (Figure 3e and data not shown). These reduction prompted us to analyse synaptic plasticity and particularly long-term potentiation (LTP), which has an important role in learning and memory.^21^ To induce LTP in cortical cultures, we used a well-described chemical stimulation protocol of forskolin plus rolipram that results in prolonged LTP (chemical LTP, cLTP).^22^ We then looked at delivery of GluR1 into synaptosomal membranes since incorporation of GluR1-containing AMPA-receptors into synapses is a major mechanism underlying the LTP.^23^ We found that treatment of cortical cultures with forskolin (50 *μ*M) and rolipram (0.1 *μ*M) persistently increases synaptosomal membrane GluR1 content by approx. 60 and 180% in empty vector and miR-34a cultures, respectively (Figure 3f). These results indicate that, although miR-34a overexpressing neurons have lower basal level of GluR1, they respond to a synaptic stimulus more efficiently than control neurons.

### Doublecortin is a new target of miR-34a

**Figure 4:**
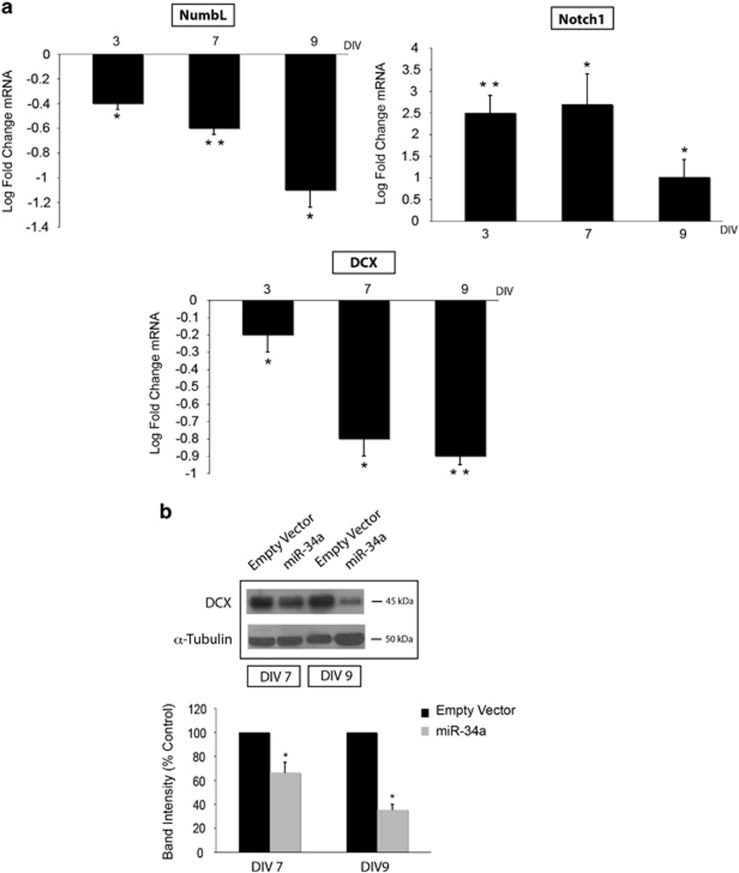
miR-34a reduces DCX expression in cortical neuronal cultures. (**a**) Real-time PCR from RNA isolated from cultures at different DIV, transduced with the empty vector or miR-34a vector. Histograms represent values plotted as log (base2) fold change to control (empty vector at the same DIV). Significant reduction is observed for NumbL transcript, while upregulation is observed for Notch1. Interestingly, a significant reduction of DCX mRNA in more mature neuronal cultures (about 30% at DIV 7, 70% at DIV 9) is observed. 18S rRNA expression was used for each sample normalization. Bars represent means±S.E.M. *P<0.05; **P<0.01 compared with control values. (**b**) Western blot analysis (top) on extract from transduced cultures (DIV 7 and DIV 9) confirms that DCX protein expression is decreased as shown by the normalized densitometric analysis (bottom). Bars represent means±S.E.M. *P<0.05 compared with control values

miR-34a acts on different mRNAs of known or potential relevance in neuronal function and plasticity including Notch1 and Numb-Like (NumbL).^7, 24^ Therefore, we evaluated the expression levels of these transcripts by real-time PCR and found a significant decrease of NumbL but an unexpected significant increase of Notch1 levels (Figure 4a). However, a similar Notch1 mRNA enhancement was observed by Fineberg *et al.*,^7^ in murine neural progenitor cells.

Because of the observed delay in the maturation of neurons overexpressing miR-34a, we next analysed the expression of Doublecortin (DCX), a neuronal cytoskeleton protein that is critical for maturation and establishment of neuronal morphology.^25, 26^ Real-time PCR and western blot analysis on extracts from miR-34a infected cultures, both showed a significant decrease of DCX expression (Figures 4a and b), which reached approx. 64% reduction in protein levels at 9 DIV (Figure 4b), peaking at 13 DIV (data not shown).

Next, we investigated whether the reduction of DCX expression could be directly caused by miR-34a targeting this transcript.

**Figure 5:**
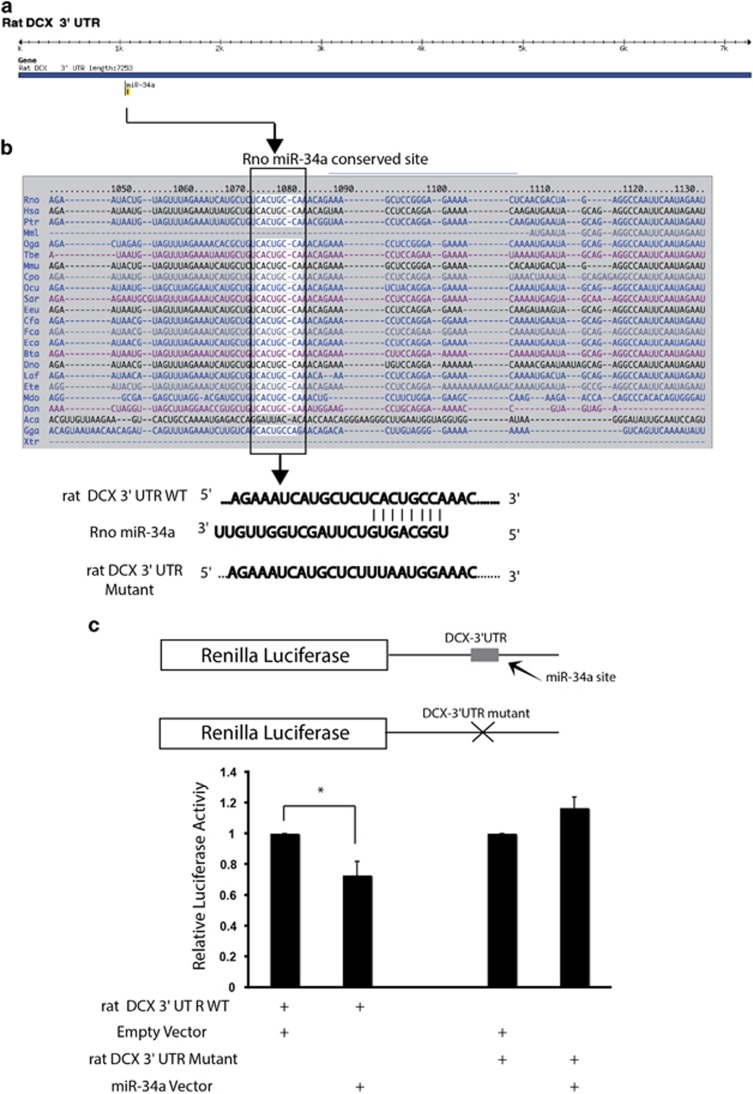
DCX transcript is a new target of miR-34a. (**a**) Schematic representation of the 3′UTR region of the rat DCX transcript showing the presence of a single binding site for the rat miR-34a as predicted by miR/target gene prediction algorithms. (**b**) A database search showing the binding site for the seed sequence of miR-34a in the 3′UTR of DCX and is highly conservation among several different species. Binding site prediction information and degree of conversation in mammalian species was obtained from TargetScan. Wild-type DCX 3′UTR annealed with the rat miR-34a and the corresponding mutant DCX 3′UTR regions are also shown. (**c**) (Top) Schematic representation of the Renilla luciferase reporter constructs in psiCHECK2 for DCX 3′UTR. (Bottom) Relative luciferase gene activity of a reporter vector harbouring the wild-type 3′ UTR of rat DCX mRNA or the mutated region downstream of a luciferase gene in the presence of rat miR-34a vector or empty vector is shown in the histogram. Co-transfections of the plasmids expressing miR-34a or the relative control plasmids along with the psiCHECK2 vector carrying the wild-type or mutated DCX 3′UTR were performed on Neuro2A, and luciferase activity was measured at 24 h after transfection. Values are mean±S.E.M. *P<0.05, compared with the respective control

We therefore took a bioinformatic approach to identify DCX mRNA potential sites as target of miR-34a. TargetScan 6.2, PicTar and miRanda were used to identify one predicted miR-34a target site in the long 3′UTR region of DCX. A schematic drawing of rat DCX mRNA and the location of miR-34a binding site are illustrated in Figure 5a. Moreover, the unique miR-34a binding site is conserved in the DCX-3′UTR from different species (Figure 5b). To confirm DCX as a potential target for miR-34a, we performed a luciferase reporter assay in N2a cells to assess transcript regulation through its 3′UTR. First, we cloned the 3′UTR of DCX containing the predicted miR-34a target site from rat brain cDNAs into a Renilla luciferase (R-luc) reporter construct (Figure 5c). Co-transfection experiments revealed that overexpression of miR-34a leads to a decrease in R-luc activity by approx. 30% with respect to control levels (Figure 5c). To further validate the interaction between miR-34a and its target DCX-3′UTR, we mutated the seed sequence of miR-34a located within the DCX-3′UTR reporter (Figures 5b and c). This mutation did not significantly change R-luc activity as compared with control, suggesting that the action of miR-34a is specific to the miR-34a seed region within the DCX-3′UTR.

Collectively, these results show that miR-34a operates as a regulator during neuronal development in part by directly targeting DCX transcript.

### miR-34a regulates progenitor proliferation and phenotypic maturation of new neurons *in vivo* in adult rat brain

We then injected miR-34a and empty rAAV expressing vectors into the cerebral lateral ventricles of rat pups at birth (P0). Injection of the virus in neonatal brain showed no signs of inflammatory reaction and, as compared with adult brain, higher penetration and a more efficient transduction of neuronal structures (Supplementary Results; Supplementary Figures S3 and S4).^27^

**Figure 6:**
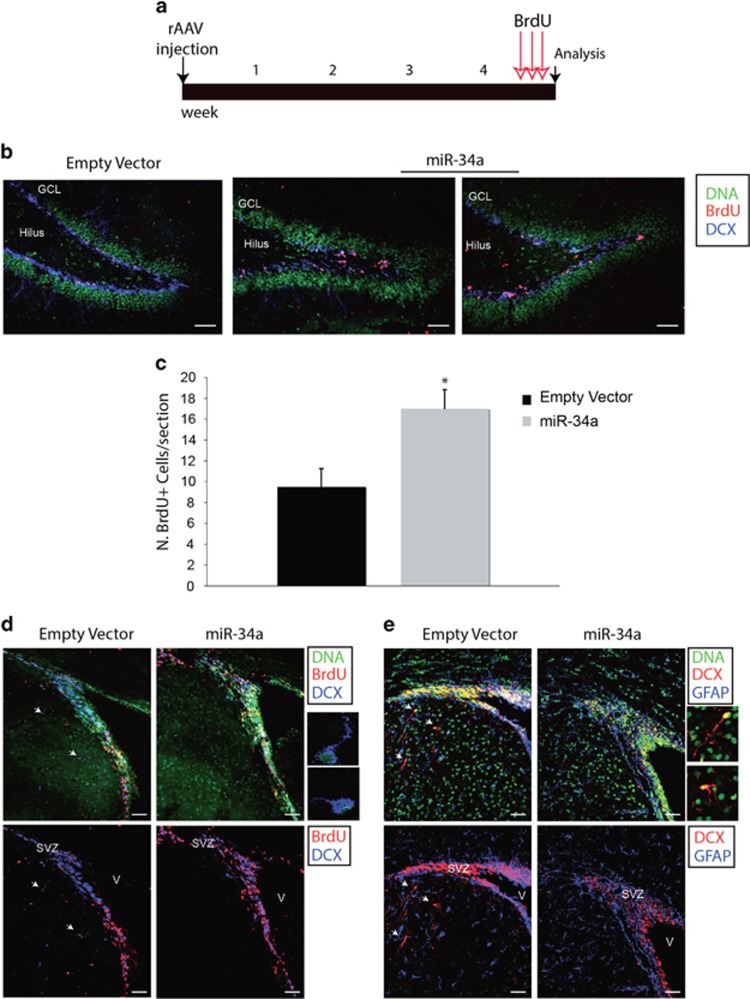
miR-34a affects neurogenesis *in vivo* and reduces neuroblast migration from the SVZ. (**a**) Schematic representation of the experimental design and time structure of the protocol to examine proliferation after rAAV injection. Animals received rAAV ventricular injection at P0. Three days before analysis, animals received three BrdU injections. Each arrow represents an injection in 1 day. Analysis was then performed at 5 weeks after the beginning of the experimental protocol. (**b**) Triple-labelled confocal images of the DG labelled for DNA (green), anti-BrdU (red) and DCX (blue), showing an increase in the number of BrdU+ cells in the subgranular zone of miR-34a-infected animals compared with empty vector-injected control animals. Often BrdU+ cells appeared grouped in small aggregates in DG of miR-34a injected animals. Scale bar: 50 *μ*m. (**c**) Quantitation of the BrdU+ cells shows a 1.8-fold increase after miR-34a overexpression. Values are mean±S.E.M. *P<0.01 compared with control values. (**d**) Confocal images of the neurogenic niches surrounding the lateral ventricles triple-labelled with DAPI (green), for BrdU (red) and DCX (blue). A higher number of BrdU+ cells in the SVZ is evident in miR-34a-injected animals as compared with controls. Moreover, a lower number of DCX+ migrating neuroblasts emanating from the SVZ are present in miR-34a-injected animals compared with controls. Arrows point on DCX+ migrating neuroblasts in control brains. Two insets show DCX+ neuroblasts with a typical morphology of migrating cells such as a small body and a leading process. (**e**) Confocal images of the region surrounding the lateral ventricles triple-labelled with DAPI (green), DCX (red) and GFAP (blue). DCX immunostaining is reduced in the SVZ of miR-34a animals as compared with controls along with a significant reduction of migrating neuroblasts expressing DCX originating from the SVZ and localized in subcortical brain regions. Arrows point on DCX+ migrating neuroblasts in control brains. Two insets show DCX+ neuroblasts with a typical morphology of migrating cells, consisting of a small ovoid cell body and a long leading process. Scale bar: 50 *μ*m

We injected one group of animals (*n*=6 for each experiment, 5 experiments) with rAAV vector expressing miR-34a, and another group with the empty vector (*n*=6 for each experiment). At 5 weeks post injection (5wpi), animals were killed and dissected brain slices were used for morphological and proliferative analysis by confocal microscopy. We analysed the proliferation of precursors in the Dentate Gyrus (DG) and Subventricular Zone (SVZ) exploiting the BrdU labelling using the following protocol (Figure 6a). rAAV-injected animals received a pulse of the mitotic marker BrdU for 3 days at the end of which they were kiled. Animals injected with rAAV-miR-34a had an increased BrdU incorporation as compared with controls, and BrdU+ cells were clustered in small isolated groups along the granule cell/hilus border (Figure 6b). Moreover, quantitative analysis of BrdU labelling within the subgranular zone revealed a 1.8-fold increase in mitotic activity (Figure 6c). Similarly, we observed a higher number of BrdU-labelled cells in the SVZ of miR-34a-transduced animals as compared with empty vector (Figure 6d).

It is known that in SVZ, stem and progenitors cells give rise to motile neuroblasts, expressing DCX, whose migration depends on this microtubule-associated protein.^28^ Interestingly, we found that miR-34a overexpressing sections show a significant reduction of DCX labelling in the progenitor cells located in the SVZ regions (Figure 6d) and a decreased number of DCX+ migrating neuroblasts (Figures 6d and e).

**Figure 7:**
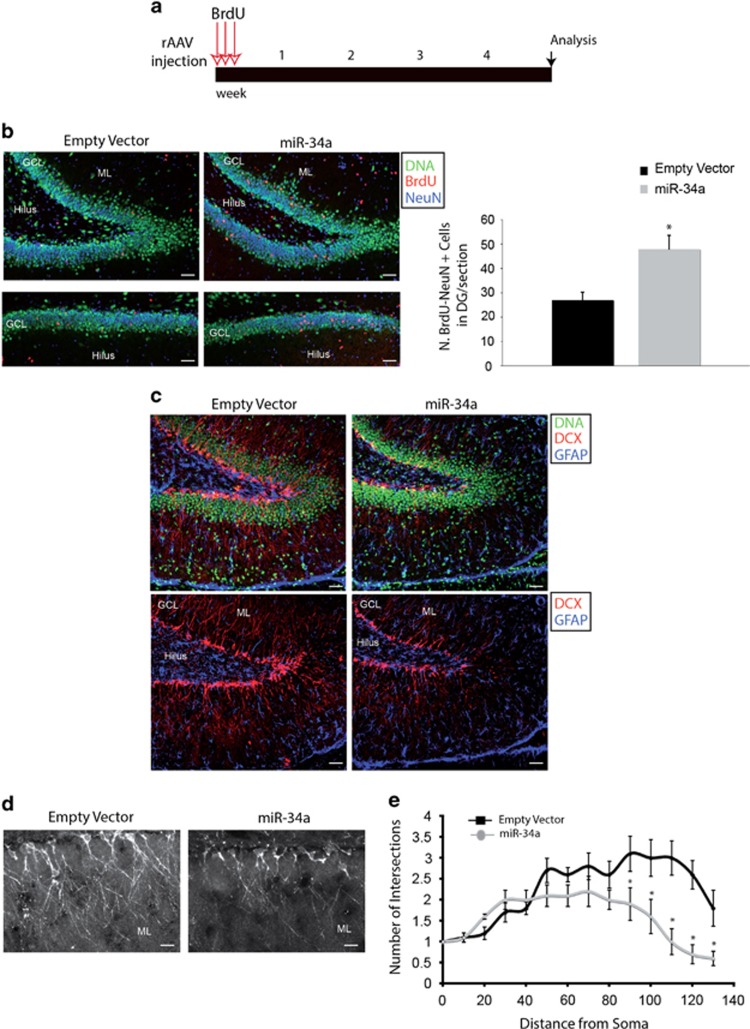
miR-34a controls *in vivo* neurite complexity of newborn granule cells of the DG. (**a**) Scheme of experimental design. Animals received rAAV ventricular injection at P0. In the same day, animals received the first BrdU injection followed by two others. Each arrow represents an injection in 1 day. Analysis was then performed at 5 weeks after the beginning of the experimental protocol. (**b**) Left panel: confocal images triple-labelled with DAPI (green), and for BrdU (red) and NeuN (blue) of adult DG from control and miR-34a-infected animals. Co-localization of BrdU staining with the neuronal marker NeuN is shown by confocal microscopy in the granule cells layer (GCL) of the DG. Note a higher number of granule neurons double-positive for BrdU and NeuN after rAAV-miR-34a injection. Lower left panel: a confocal image of the lower blade of the adult DG showing granule neurons immunolabelled for both NeuN and the anti-BrdU. Scale bar: 50 *μ*m. Right panel: comparison of the number of BrdU+/NeuN+ cells within the DG shows a 1.8-fold increase in miR-34a-injected animal as compared with controls. Bars represent means±S.E.M. *P<0.01 compared with control values. (**c**) Examples of confocal images triple-labelled with DAPI (green), and for DCX (red) and GFAP (blue) of adult DG from control and miR-34a-infected animals. DCX staining appears reduced in rAAV-miR-34a injected animals. No alterations are observed with GFAP immunostaining. Scale bar: 50 *μ*m. (**d**) Visualization of newly generated neurons in sections of the DG labelled with DCX from infected animals. DCX labelling identifies neuronal cell bodies and vertically oriented dendrites of maturing neurons in the DG. DCX immunostaining is reduced and neurites appear less complex and more confined into the GCL. Scale bar: 50 *μ*m. (**e**) Sholl's concentric spheres analysis performed on maturing granule neurons reveals an altered dendritic morphology as compared with control neurons. Control neurons have more intersections indicating a higher neurite complexity. Interestingly, most of these control neurons have processes that extend deeper into the molecular layer (ML) of the DG, suggesting a higher capacity of dendritic outgrowth. Graph represents means±S.E.M. *P<0.05 compared with control values

To determine the long-term fate of mitotically active cells in the DG of miR-34a animals, we administrated three pulses of BrdU starting at the day of viral injection (P0) and then killed animals at the fifth week after infection (Figure 7a). Coronal brain sections stained for BrdU and NeuN showed a significant number of BrdU+ cells which subsequently migrated into the granule cell layer and differentiated into neurons with mature granule cell features (Figure 7b, left panel). Quantitative analysis of BrdU+/NeuN+ cells showed a 1.8-fold increase in cell number per section in the DG of miR-34a, as compared with empty vector animals (Figure 7b, right panel). All together these results confirm that miR-34a acts as a mitogen for precursors both *in vivo* and *in vitro.*

We then examined whether miR-34a high levels of expression could affect the dendritic growth of newly born neurons in the DG, by immunostaining with DCX. In both injected group of animals, we found that DCX+ cells were expressed throughout the DG with their dendrites extended out to the molecular layer of the DG (Figure 7c). Interestingly, sections from miR-34a animals showed a reduction of intensity of DCX immunostaining (32% reduction), as compared with empty vector sections. Moreover, neurons in the young adult DG of miR-34a animals exhibited a lower dendritic complexity and a shorter distance reached by the dendritic tree into the molecular layer of the DG (maximum length from the soma, 111±22.5 *μ*m) as compared with controls (maximum length from the soma, 144±6.5 *μ*m) (Figures 7c and d). The concentric circle analysis of Sholl confirmed a remarkable change in dendritic morphology and pattern of growth of the newly generated neurons (Figure 7e).

### miR-34a overexpression *in vivo* influences emotionality and cognitive abilities

To investigate whether alterations of hippocampal neurogenesis and neuronal maturation, induced by miR-34a, were associated with specific changes in cognitive and emotional behaviour, experimental subjects were tested for learning and memory in a spatial navigation test, the Morris water maze (MWM) and for social anxiety in the Social Interaction Test (SIT).^29^

**Figure 8:**
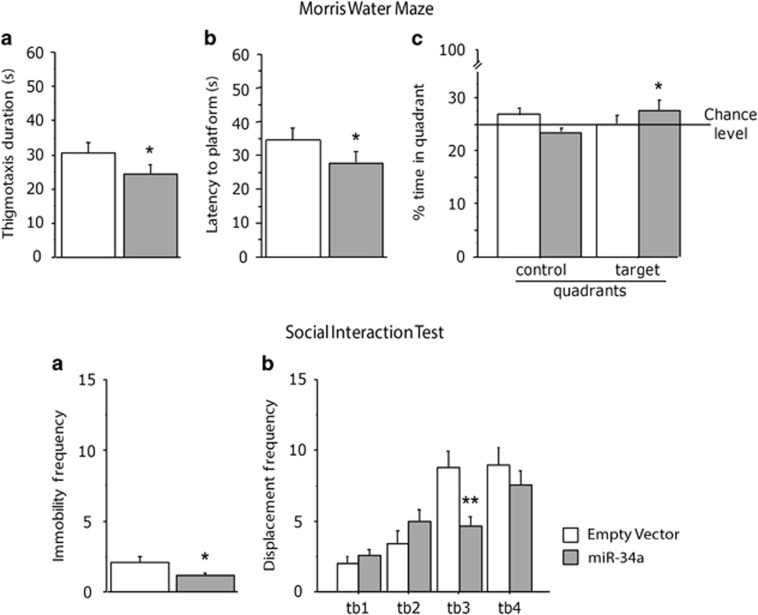
miR-34a overexpression, mediated *in vivo* by rAAV, improves cognitive abilities and reduces anxiety. Behavioural tests were performed on adult male rAAV-infected rats (5 weeks old). (*Upper panel*) Results from the MWM spatial navigation task indicate that miR-34a rats were characterized by a reduced thigmotaxis upon introduction in the novel environment (pool), and a behaviour suggesting reduced anxiety (**a**). In addition, when compared with controls, they showed better learning abilities in the acquisition phase (**b**) and memory retention in the probe trial (**c**). (*Lower panel*) Results from the SIT indicate a reduced emotionality, as miR-34a animals were more active (**a**) and characterized by reduced de-arousal behaviours during the social interaction test (**b**). The maximum length of social encounters was 20 min, each session was subdivided into 4 time blocks (tb). Data show mean±S.E.M (MWM, *n*=8 for each experimental group; SIT, *n*=10 for each experimental group). *P<0.05: **P<0.01

As for the MWM (miR-34a=10; empty vector=10), upon introduction in the pool, miR-34a rats were characterized by decreased thigmotaxis, suggesting a reduced emotionality in a novel environment (pool) (F(1, 14)=5.410; *P*=0.0356, Figure 8a). In addition, and more importantly, during the acquisition phase, they were characterized by a reduced latency to reach the hidden platform (F(1, 14)=4.511; *P*=0.0520, Figure 8b), suggesting improved learning abilities. When the platform was removed from the pool to assess memory retention, miR-34a rats were successful in spending more than 25% of the allotted time (chance level) searching the platform in the target quadrant, whereas control rats did not reach the chance level showing a poorer cognitive performance (F(1,14)=3.225; *P*=0.0334; *post hoc* comparison: target quadrant, miR-34a *versus* empty vector, *P*<0.05, Figure 8c). Four subjects were discarded from the analysis (two empty vector and two mir-34a-treated rats) because they never reached the platform during the acquisition phase.

Results from the SIT (miR-34a=10; empty vector=10), overall showed that miR-34a rats were more active, being characterized by a reduced frequency of immobility bouts (*Z*=−1.965; *P*=0.0494, Figure 8a); on the contrary, immobility duration did not differ between the two groups of animals *(Z*=−1.512; *P*=0.1306). In addition, miR-34a animals also performed a reduced amount of displacement behaviours (F(1,18)=1.886; *P*=1.886, Figure 8b) suggesting a reduced social anxiety; duration of this behavioural category did not differ between the two groups (*Z*=−0.680; *P*=0.4963). As for the remaining behavioural items miR-34a and empty vector treated subjects did not differ from each other (frequencies: F(1,18)=0.284; 0.005; 0.962; *P*=0.6007; 0.9423; 0.3397; durations: F(1,18)=0.277; 0.029; 1.680; *P*=0.6053; 0.8656; 0.2113, respectively for affiliative, explorative and play soliciting behaviours).

Overall, behavioural analysis demonstrated that miR-34a overexpression improves cognitive abilities in the MWM performance and reduces anxiety in a social context in adult rats.

## Discussion

Here we show for the first time that miR-34a has an important modulatory role in neurogenesis and maturation of newly born neurons both *in vitro* and *in vivo*. In particular, we present evidence that miR-34a overexpression results in: (i) increased proliferation of precursor cells; (ii) inhibition of neurite branching and delayed maturation of developing neuronal cells; (iii) altered physiological features of mature neurons; (iv) improvement in behavioural outcomes. Importantly, we identified DCX as a new miR-34a target whose downregulation might be in part responsible for the effects mediated by miR-34a in brain.

miR-34a is well recognized as a tumour suppressor in brain and many other tissues.^30^ However, several studies also point out a progrowth function and highlight variable miR-34a effects depending on cell type.^18^ For instance, miR-34a knockout mice show a reduced number of precursor proliferating cells in the DG.^9^ Moreover, p73-deficient mice, which display reduced levels of miR-34a, present important malformations of telencephalon and reduced adult neurogenesis.^8, 31^ In agreement with these findings, we found that high levels of miR-34a expression induce an increased number of proliferating precursors both *in vitro and in vivo* confirming its distinct growth effects depending on the stimuli and cellular context.

Previous *in vitro* studies showed that miR-34a has profound effects on the arborization of cortical neurons.^8, 9^ Our data confirm and extend *in vivo* the observation that miR-34a has an important role in the development of neuritic arborization and growth of newly generated neurons. To our knowledge, this is the first study in which the knock-in effects of miR-34a were evaluated in the brain and the first demonstration of an *in vivo* role of miR-34a in the control of maturation and neuronal morphology in post-mitotic developing neurons.

Moreover, it is noteworthy that miR-34a is present in exosomes released in the medium of neuronal cultures since it may indicate that miR-34a can affect cells both cell-autonomously and non-cell-autonomously. This would determine a higher diffusion and spreading of its modulatory activity from expressing cell to the neighbouring cells.

The reduced dendritic branching and delayed maturation we observed might be due to deficits in the neuronal transport mechanism or abnormalities in cytoskeleton structures. Interestingly, we found a decreased expression of the cytoskeletal protein DCX both *in vitro* and *in vivo*. A link between DCX and miR-34a was previously suggested by Genovese *et al.* ^32^ in a network of miRs in which miR-34a acts as a master regulator.

We have identified DCX as a new miR-34a target. Using bioinformatic databases, we have identified one highly conserved binding site in the 3′UTR of DCX and confirmed, by luciferase assay, that miR-34a can bind to it and directly regulate DCX expression. Nevertheless, we cannot exclude that other factors may mediate the reduction of DCX by miR-34. It is noteworthy that a striking feature of DCX mRNA is a 7.9-kb long 3′UTR,^25^ containing AU-rich regulatory elements, which further point towards post-transcriptional regulatory mechanisms.

DCX has been associated with neuronal differentiation and functionally linked to cell migration and other aspects of maturation, including dendritogenesis and synaptogenesis.^33^ Modification of expression levels of DCX alters the arborization of dendrites and DCX depletion retards the development of cultured hippocampal neurons, maintaining them in an immature state.^33, 34^ Another important DCX function is the control of cellular migration during embryonic and postnatal brain development.^35^ Indeed, we found that miR-34a overexpression affects migration of neuroblasts immediately adjacent to the SVZ. Because miR-34a is involved in cellular motility of neural precursors,^36^ it is possible that this function is mediated by DCX which in turn would control the cytoskeletal rearrangements necessary for cellular motility in rodent brain. However, we cannot exclude that some effects mediated by miR-34a overexpression are due to other cellular players. Interestingly, we found that transcript levels of Notch1 are elevated and those of NumbL are decreased. Notch1 and NumbL are critical players in the complex regulation of neurogenesis, balancing proliferation and differentiation.^24^ It would be important to determine whether, depending on the cellular context and the abundance and availability of target transcripts, the DCX pathway becomes dominant and prevalent on other possible effectors of miR-34a action.

In agreement with previous studies, we found that miR-34a overexpression in developing neurons causes a reduced expression of several synaptic proteins,^9^ and receptor subunits, some of which are not among the predictable targets of miR-34a. Along with this, we showed a decrease in NMDA-evoked current density further suggesting a delayed maturation of miR-34a overexpressing cells. Surprisingly, neurons with these features responded more efficiently to a synaptic stimulus, with respect to control neurons. A possible explanation resides in previous studies demonstrating that immature neurons have an increased intrinsic excitability and plasticity, which distinguish them from the less plastic older population.^37^ Due to their physiology and low connectivity, these immature neurons are responsive to a wide range of inputs. Thus, in our condition, it is possible that immature miR-34a overexpressing neurons show enhanced plasticity because of specialized membrane properties and different rules for LTP expression. Indeed, Schmidt-Hieber *et al.*^38^ suggested that newly generated neurons, having a threshold for LTP induction lower than control neurons, express unique mechanisms to facilitate synaptic plasticity that may be important for the formation of new memories. However, we cannot exclude, as suggested by Agostini *et al.*,^9^ that miR-34a overexpression might enhance neuronal plasticity also by preferentially disrupting inhibitory inputs.

New neurons are added continuously to certain areas of the adult brain such as the hippocampus, implicated in learning/memory and emotional behaviours.^39^ In the adult hippocampus, the increment of immature young cells may influence hippocampal functioning.^40^ Because of their ability to form new synapses rapidly, newly generated neurons can form a greater number of new connections than resident mature cells thus playing an important role in hippocampal-dependent learning. We found that miR-34a increases *in vivo* progenitor proliferation and alters phenotypic maturation of new neurons in the DG of adult rats. Moreover, we found that rats overexpressing miR-34a in the brain have better learning abilities in the acquisition phase and memory retention in MWM test and a reduced emotionality in the SIT. Thus, miR-34a could influence learning and anxiety-related behaviours by enhancing production of new neurons and modulating their maturation stage. Consistently, recent reports have implicated miR-34 family in regulating genes that mediate the behavioural changes in response to stress.^41, 42^

miR-34 increases with age in *C. elegans* and *D. melanogaster*.^43, 44^ In mammals miR-34a orthologue also increases with age^5, 45^ and is misregulated in degenerative diseases.^45, 46^ Moreover, inactivation of miR-34 expression has been recently shown to lead to accelerated neurodegeneration and ageing in *D. melanogaster,*^44^ whereas in vertebrates its elevation has been suggested to be either protective or contribute to age-associated events.^46, 47^ In addition, miR-34a expression is upregulated in brain tissue,^48^ blood mononuclear cells,^49^ and cerebrospinal fluid (CSF),^50^ of Alzheimer's disease patients.

On the basis of these observations, it is possible that miR-34a, harbouring cell cycle-related functions in dividing and differentiating cells, may have evolutionary acquired functions to silence genes that promote age-associated decline and age-related diseases. Thus, the modulation of miR-34a levels could be investigated as a potentially useful strategy to counteract aging and neurological disorders.

## Materials and Methods

### Animals

Adult time-mated female Wistar rats were purchased from Harlan Laboratories (Indianapolis, IN, USA). The authors certify that all the experimental protocols used in the present study were in compliance with the European Guide for the Care and Use of Laboratory Animals and institutional guidelines and with the Italian legislation on animal experimentation (Decreto L.vo 116/92).

### Expression constructs

See Supplementary information.

### AAV production, transduction and *in vivo* injection

rAAV (rAAV1/2) mosaic vectors containing a 1 : 1 ratio of AAV1 and AAV2 capsid proteins with AAV2 inverted terminal repeats (ITRs) were generated by crosspackaging as previously described.^51^

Briefly, the packaging HEK293 cells were cultured in DMED media (Invitrogen, Carlsbad, CA, USA) supplemented with 10% fetal bovine serum, 1 mM glutamine, 100 Units/ml penicillin and 100 *μ*g/ml streptomycin. HEK293 cells were transfected with the AAV vector plasmid, the AAV1 and AAV2 helper plasmids and mini-adenovirus helper plasmid by standard calcium phosphate transfection methods.^51^ Forty-eight hours after transfection, cells were harvested and the vector purified using heparin affinity columns (Sigma, St. Louis, MO, USA). The purity was assessed by running 10 *μ*l of the viral purification on a Coomassie protein gel. Genomic titre were determined using quantitative real-time PCR (Stratagene, La Jolla, CA, USA), using primers designed to WPRE. Generally, rAAV purification titre was estimated to vary from 1 to 2 × 10^8^ particles/*μ*l, as obtained by quantitative real-time PCR.

For *in vitro* experiments, after 3 h, when cells had attached to the substrate, 1 or 2 *μ*l of the rAAV viral particles was added into the culture medium, resulting into visual EGFP signal after 3–4 days and almost 100% transduction efficiency.

For *in vivo* experiments, postnatal day 0 rat pups (P0) were injected as described by Li and Daly.^27^ Briefly, pups were cryoanesthetized and injected with 2 *μ*l purified rAAV stock into each lateral ventricle (1 mm posterior to bregma and 2 mm lateral to the sagittal suture) using a 10-*μ*l Hamilton syringe. Individual experiments were performed on pups from the same litter and repeated on pups of different litters. Animals were tattooed on the footpads to identify the groups injected with the rAAV-empty vector or rAAV-miR-34a. Behavioural training began at the end of the 4 weeks after vector infusion when transgene protein expression had peaked to remain at stable levels. All tests were conducted by an observer blinded to the treatments.

### Primary cortical neuron culture

To obtain embryonic cortical neurons, time-mated Wistar rats were euthanized by CO_2_ and cervical dislocation. Cells were were prepared with slight modifications from the protocol proposed by Xu *et al.* ^52^ Cortical tissues of rat embryos (E15) were dissected and dissociated by trypsin treatment followed by trituration. After removal of trypsin, neurons were seeded onto 35- or 100-mm-diameter Petri dishes coated with poly-L-lysine (0.5 mg/ml, Sigma), plated at the density of 600 000 cells for imaging experiments or at a density of 800 000 cells in a 35-mm plate for biochemical analysis. For synaptosomal purifications, neurons were plated at a density of 5 × 10^6^ cells in 100-mm-diameter plates. The cultures were maintained in Neurobasal supplemented with B27 (Invitrogen), 1 mM sodium pyruvate, 2 mM glutamine and antibiotics in a humidified incubator at 37 °C and 5% CO_2_. At day 5 of culture, 25% of freshly made complete medium was added without Arabinofuranosyl Cytidine (AraC).

For proliferative experiments, 5-bromo-3′-deoxyuridine (BrdU) (1 : 100 solution, Invitrogen), was added to cultures 1 day *in vitro* (DIV 1) after plating and trasduction (DIV 0). Cultures at different experimental time points were then fixed, processed for immunofluorescence, counterstained with DAPI. For each experimental time point, mitotic cells (identified by chromosome condensation by DAPI), BrdU-labelled cells and apoptotic cells (picnotic nuclei) were counted and the percentage of proliferating and apoptotic cells was calculated.

For chemical LTP stimulations, neuronal cultures (DIV 7 and DIV 9) were first incubated in ACSF (125 mM NaCl, 2.5 mM KCl, 1 mM MgCl_2_, 2 mM CaCl_2_, 33 mM D-glucose, and 25 mM HEPES pH 7.5) for 30 min at room temperature, followed by stimulation with 50 *μ*M forskolin (Sigma) and 0.1 *μ*M rolipram (Calbiochem, San Diego, CA, USA) in ACSF (no MgCl_2_). After 10 min of stimulation, neurons were replaced in regular ACSF and then subjected to subcellular fractionation.

### Real-time PCR

See Supplementary information.

### Subcellular fractionation of rat brain tissues and primary cortical neurons

Rat neuronal cultures were subjected to fractionation as previously described,^53^ with minor modifications. Dounce homogenates of the pellets in ice-cold TEVP buffer (containing 10 mM Tris-Cl pH 7.5, 320 mM sucrose, proteases inhibitor cocktail, phosphatases inhibitor cocktail) were centrifuged at 1000 × *g* to remove nuclei and large debris (P1). The supernatant (S1) was centrifuged at 10 000 × *g* to obtain a crude synaptosomal fraction (P2) and subsequently was lysed hypo-osmotically and centrifuged at 25 000 × *g* to pellet a synaptosomal membrane fraction (LP1). Protein concentration was determined using microBCA assay (Pierce, Rockford, IL, USA) and 30 *μ*g of LP1 was subjected to SDS-PAGE.

### Protein lysis and western blot

For protein expression analysis, primary cortical neurons transduced with purified rAAV vectors were detached by pipetting and cellular pellets were lysed in RIPA buffer: 150 mM NaCl, 10 mM Tris-HCl, 1 mM EDTA, and 1% Triton X-100 and protease inhibitors (Sigma), 1 mM PMSF pH 7.4. Samples were resolved in SDS-PAGE gels with different percentages. Western blot analysis was performed as previously described.^54, 55^

The following antibodies were used: mouse anti-alpha-tubulin 1 : 1000 (Santa Cruz Biotechnology, Santa Cruz, CA, USA), mouse anti-beta-actin 1 : 1000 (Sigma); anti-p53 1 : 500 (Santa Cruz Biotechnology); rabbit anti-DCX 1 : 500 (Santa Cruz); rabbit anti-PSD95 1 : 1000 (Cell Signalling, Beverly, MA, USA), rabbit anti-NMDAR1 1 : 100 (Chemicon), rabbit anti-NMDAR2A/B 1 : 500 (Chemicon), mouse anti-synaptophysin 1 : 500 (Synaptic Systems, Goettingen, Germany), rabbit anti-synaptotagmin 1 : 500 (Sigma) anti-GluR1 1 : 1000 (Upstate, Charlottesville, VA, USA). The quantitation of protein expression was determined after normalization to tubulin or actin by measuring the optical density of respective band blots using the Quantity One software (Bio-Rad, Hercules, CA, USA).

### Exosome isolation

Medium from 8-day-old primary cultures (4–5 × 10^7^ cells) was harvested and cleared of debris by two successive centrifugation steps (2000 × *g* for 10 min and 20 000 × *g* for 20 min). Membranes were recovered from the cleared medium by centrifugation for 45 min at 100 000 × *g*. They were resuspended in Trizol (Invitrogen) for RNA isolation as described in Faure *et al.*^56^

### Electrophysiology

Membrane currents were recorded from the cell soma of cortical cells at 8–11 days. The whole-cell configuration of the patch-clamp method was used. Recordings were performed with pipettes pulled from capillary tubes with a Narishige micropipette puller (tip resistances 4–5 MΩ).

All recordings were performed at room temperature (22–24 °C) and obtained using an Axopatch 200B amplifier (Molecular Devices, Sunnyvale, CA, USA). pCLAMP 8 software was utilized for the data acquisition system. The signals were filtered at 2–10 KHz and digitized at 20–50 KHz. After the establishment of a gigaseal, the pipette resistance and capacitance were compensated electronically and the cells were accepted for study only if these parameters remained stable.^57^ The cells chosen for the electrophysiological recordings looked like pyramidal neurons and represented more than 80% of the neuronal population in the culture. Cell capacitance was measured for each cell from the amplifier and from transient currents produced by 10 mV hyperpolarizing voltage steps. The values of the peak current were normalized to cell capacitance. Different solutions were applied by gravity using small tubes (diameter <1 micron), which were placed at near the patched cell using a fast perfusion system (SF-77B Warner Ins., Hamden, CT, USA). During the experiment, cells were continuously perfused with a control solution. Experiments were carried out in a bath medium that contained 120 mM NaCl, 3 mM KCl, 2 mM CaCl_2_, 2 mM MgCl_2_, 20 mM glucose and 10 mM HEPES (pH 7.3 with HCl). The whole-cell recording pipette used for recording the membrane currents contained 130 mM CsCl, 20 mM tetraethylammonium chloride, 5 mM EGTA, 10 mM glucose, 10 mM HEPES, 1 mM MgCl_2_, 0.24 mM CaCl_2_ and 2 mM ATP (pH 7.3 with CsOH). The NMDA (0.2 mM) was dissolved in the bath medium, which was Mg^2+^ free and contained 3 *μ*M of glycine.

### Immunofluorescence

Neurons in cultures grown on poly-L-lysine-coated glass coverslips were fixed with 4% paraformaldehyde/phosphate-buffered saline for 20 min at 37 °C, washed for 5 min with phosphate-buffered saline, permeabilized with 0.2% Triton X-100 in phosphate-buffered saline for 3 min and washed three times for 5 min with phosphate-buffered saline. For BrdU labelling, fixed neurons were incubated with 2 N HCl for 30 min at room temperature to denature DNA, followed by 0.1 M sodium tetraborate 5 min and then washed several times in PBS, before proceeding with the primary antibody incubation.

Cells were then processed with primary and secondary antibodies and counterstained with DAPI. The following primary antibodies were used: mouse anti-BrdU (Santa Cruz Biotechnology) diluted 1 : 80; mouse anti-MAP2 (Sigma), diluted 1 : 500; rabbit anti-GFAP (Chemicon) diluted 1 : 1000; anti-NeuN (Millipore, Darmstadt, Germany) diluted 1 : 400; mouse anti-beta-tubulin III (Chemicon) and goat anti-DCX (Santa Cruz Biotechnology) were diluted 1 : 250. All antibodies were diluted in phosphate buffer containing 3% bovine serum albumin and 0.05% Tween-20 and incubated 1 h at at 37 °C. After rinsing, the coverslips were incubated with fluorescently-labelled secondary antibodies (1 : 250) (Jackson Immunoresearch Laboratories, West Grove, PA, USA and Molecular Probes, Eugene, OR, USA) for 30 min at room temperature. After a thorough rinse, coverslips were mounted and analysed by confocal microscope.

### Immunofluorescence on brain tissues

Immunofluorescence on brain tissues was performed as previously described.^58^ Free-floating sections (35 *μ*m thickness) for BrdU staining were pretreated with 2 N HCl for 30 min at room temperature to denature DNA, followed by 0.1 M sodium tetraborate 5 min and then washed several times in PBS. Sections were then blocked for 1 h in PBS with 0.2% Triton X-100 containing 10% donkey serum. Primary antibodies were incubated for 24 h at 4 °C.^19^ The primary antibodies were used at the following concentrations: goat polyclonal anti-DCX (Santa Cruz, CA, USA), 1 : 200; mouse monoclonal anti-neuronal nuclei NeuN (Millipore), 1 : 400; mouse anti-GFP (Dako, Glostrup, Denmark) diluted 1 : 250; mouse anti-MAP2 (Sigma), diluted 1 : 500; rabbit anti-GFAP (Dako) diluted 1 : 1000; rabbit anti-iba1 (Biocare Medicine, Concord, CA, USA) diluted 1 : 500; mouse anti BrdU (Santa Cruz Biotechnology), mouse monoclonal anti-GFP (Millipore), 1 : 250. Secondary antibodies against appropriate species were incubated for 1 h at 37 °C (1 : 250) (Jackson Immunoresearch Laboratories and Molecular Probes). Fluorescently stained sections were coverslipped with glycerol in PBS containing an anti-fading. Fluorescently immunolabelled sections were analysed on an Olympus (Tokyo, Japan) FV1000 confocal laser scanning microscope. Confocal Z sectioning was performed at 0.8 *μ*m intervals using a × 60 (NA=1.42) oil-immersion objective for single and double scanning. Images were acquired and a Z-stack was reconstructed using the Olympus software (Tokyo, Japan), cropped, adjusted and optimized in Photoshop 9.0. Other images of fluorescently immunolabelled sections were acquired using a Nikon (Tokyo, Japan) microscope.

### *In vivo* neurogenesis analysis

The thymidine analogue BrdU (Invitrogen) was used *in vivo* to label proliferating cells. Two different protocols of injections were performed to estimate the proliferation rate, cell fate determination and survival. In one case, rat pups (*n*=6 per group) received three daily consecutive sub-dermal injections of BrdU (20 mg/kg) (Invitrogen), once every 12 h, starting from the day of rAAV injection. In a second protocol, young rats (*n*=6 per group) after 4 weeks from the rAAV infection, received three consecutive BrdU (80 mg/kg) intraperitoneal injections, once every 12 h. Rats were killed 24 h after the last BrdU administration and perfused for further tissue analysis.

To estimate the number of BrdU-labelled cells present in the SVZ and dentate gyrus, we sampled every three sections throughout the rostrocaudal extent of each structure from the rAAV-empty vector and rAAV-miR-34a stained sections labelled for BrdU. Each of the regions quantified was defined based on anatomical and stereotaxic coordinates. Quantification of BrdU^+^ cells was performed using the ImageJ software (NIH, USA) on confocal images of a specific brain region. For each region, the results were reported as the mean number of BrdU^+^ cells per section. The number of BrdU+ cells was then averaged to represent each brain.

### Sholl analysis

Six to nine EGFP^+^ cortical rat neurons were chosen from cell culture and scanned using a FluoView 1000 (Olympus) ( × 40, 1.3 NA or × 60, 1.42 NA). Similarly, for analysis in the DG of infected animals, six to nine cells expressing DCX cells (per animal) with vertical oriented dendrites extending into the dentate molecular layer were traced in their entirety. Images of collapsed z-stacks were imported into NIH ImageJ, and dendritic complexity was analysed from 8-bit images by using the ImageJ Sholl Analysis plug-in (http://rsb.info.nih.gov/ij/). Total dendritic length and number of intersections of each individual neuron were analysed. The centre of all concentric circles was defined as the centre of the cell's soma. The parameters used were starting radius (10 *μ*m), ending radius (300 *μ*m from the centre) and interval between consecutive radii (10 *μ*m).

### Behavioural tests

#### Social Interaction Test

The night before the test all subjects (MiR-34a=10; empty vector=10) were placed individually in a holding cage, a procedure known to stimulate social interactions.^59, 60^ Experimental subjects were placed in a novel cage, identical to the holding cage, with an unfamiliar conspecific of the same strain, weight and sex that had been previously isolated (standard opponent). The maximum length of social encounters was 20 min; frequency and duration of environmental exploration (sum of crossing, sniffing and rearing), social investigation (sum of body, nose and ano-genital sniffing and follow), affiliative behaviours (allogrooming, social rest and social inactive) and displacement/de-arousal behaviours (sum of digging and self-grooming) were scored. For a complete description of behavioural items, see Circulli *et al.*^59^ and Berry *et al.*^60^

#### Morris Water Maze

Experimental subjects (miR-34a=10; empty vector=10) underwent a 1-day MWM test.^61^ In particular, rats were introduced in a Plexiglas circular pool (180 cm, diameter) ideally subdivided into four quadrants and filled with water (24–26 °C) and underwent six acquisition trials to learn to locate the position of a platform hidden below the water surface. Each trial was spaced from the other by a 45-min interval and had 60-s duration (cutoff time) during which animals freely swam until they found the platform or until cutoff. Ninety minutes following the last training trial a 60-s probe trial memory test was performed by removing the platform from the maze. The following behaviours were scored: thigmotaxis (i.e., swimming along the pool border, a behaviour indicative of risk assessment), latency to reach the platform (acquisition phase); time spent in the acquisition quadrant compared with other quadrants (probe trial memory test). Behaviour was analysed with a dedicated software (Ethovision, Noldus, Wageningen, The Netherlands).

### Luciferase analysis

To examine the knockdown potencies of miR-34a on the 3′UTR region of rat DCX, we constructed reporter genes with the psiCheck2 vector (Promega, Madison, WI, USA). The 3′ UTR of rat Dcx (a PCR fragment around 1000 bp length) was cloned downstream of the Renilla luciferase gene using the Not1-Xho1 sites of the vector. Neuro2a (N2a), a mouse neuroblastoma cell line, was used for luciferase assay. Briefly, the cells were grown in Dulbecco's modified Eagle's medium (Invitrogen) supplemented with 10% fetal bovine serum (Invitrogen), 100 U/ml penicillin and 100 *μ*g/ml streptomycin at 37 °C in 5% CO_2_-humidified chamber. The day before transfection, cells were trypsinized, diluted with culture medium without antibiotics, and seeded into 6-well culture plates to reach a 70% confluency. Lipofectamine-2000 transfection reagent (Invitrogen) was performed according to the manufacturer's instructions. For assessment of the knockdown potency of miR-34a, the transfection on N2a was conducted with the following plasmids: 500 ng psiCheck2- 3′UTR plasmid and 2 *μ*g rAAV-miR-34a or rAAV empty vector. At 24 h after transfection, cells were lysed and analysed for luciferase activity using the Dual-GloTM Luciferase Assay System (Promega). Renilla luciferase values were normalized to control firefly luciferase levels (transcribed from the same vector but not affected by the 3′UTR tested), (Renilla luciferase/firefly luciferase), and averaged across three-well repetitions per condition. Data are the average of three separate transfections performed on different days with different passage numbers of cells.

### Statistical analysis

Statistical significance was assessed by unpaired two-tailed Student's *t*-tests. A probability level of less than 0.05 was accepted as statistical significance. Degrees of statistical significance are presented as *P<0.05 and *P<0.01. Data in the text and figures were generally expressed as means±S.E.M. For electrophysiological experiments, statistical analyses were performed using SPSS 11.0.0 for Windows (SPSS Inc., Chicago, IL, USA). All results were expressed as mean±S.D., with *n* the number of tested cells. The significance of the effect was performed by unpaired two-tailed Student's *t*-tests. The significance level was set to 0.05. Behavioural data were analysed using parametric analysis of variance (ANOVA) with treatment as between-subjects factor and time blocks as within-subject repeated measures both for SIT and for MWM. Since data did not follow a normal distribution, a Mann and Whitney *U* test was applied to analyse the immobility behaviour in the SIT. *Post hoc* comparisons were performed using the Tukey's test.

## Abbreviations

AAV: adeno-associated virus
BrdU: 5-bromo-3′-deoxyuridine
DAPI: 4′,6-diamidino-2-phenylindole
DCX: Doublecortin
DG: dentate gyrus
DIV: day *in vitro*
EGFP: enhanced green fluorescent protein
GCL: granule cell layer
GFAP: glial fibrillary acidic protein
GLUR1: glutamate receptor 1
ITRs: inverted terminal repeats
miR-34a: microRNA-34a
ML: molecular layer of the dentate gyrus
MWM: Morris Water Maze
NMDAR: *N*-methyl-D-aspartate receptor
Notch1: Notch homologue 1 translocation-associated (*Drosophila*)
NUMBL: Numb-like protein
PSD95: postsynaptic density protein 95
SIT: social interaction test
SVZ: subventricular zone
3′UTR: 3′untranslated region
WPRE: Woodchuck post-transcriptional regulatory element
