# Supplementary material for: miR-34a regulates cell proliferation, morphology and function of newborn neurons resulting in improved behavioural outcomes
Source: Cell Death Dis. 2015 Jan 29;6(1):e1622–. doi: 10.1038/cddis.2014.589 (PMC4669781; doi:10.1038/cddis.2014.589)
Supplement: Supplementary information [file cddis2014589x1.pdf]

Supplementary Information

Title:

miR-34a regulates cell proliferation, morphology and function of newborn neurons resulting in improved behavioural outcomes

Cristiana Mollinari 1-2, Mauro Racaniello 2, Alessandra Berry 2, Massimo Pieri 3-4, Maria Chiara de Stefano 2, Alessio Cardinale 5 Cristina Zona 4, Francesca Cirulli 2, Enrico Garaci 5*, Daniela Merlo 2*

1 Institute of Translational Pharmacology, National Research Council, Rome, Italy

2 Department of Cell Biology and Neuroscience, Istituto Superiore di Sanità, Rome, Italy

3 Department of Experimental Medicine and Surgery, University of Rome "Tor Vergata", Italy

4 Department of System Medicine, University of Rome "Tor Vergata"

5 IRCCS San Raffaele Pisana, Rome, Italy

*** Corresponding Authors**

Content:

Supplementary Results

Supplementary Material and Methods

Supplementary Figures S1-S4

Supplementary Table S1

Supplementary References

Supplementary Results

rAAV-mediated miR-34a overexpression in vivo is widespread and neuron-specific

rAAV can transduce both replicative and non-replicative cells, such as post-mitotic neurons of the central nervous system (CNS) in a highly efficient and stable manner without inducing cytotoxicity or cellular immune response.[1, 2, 3]

We injected miR-34a and empty AAV expressing vectors into the cerebral lateral ventricles of rat pups at birth (P0). We verified that no signs of tissue damage were induced following brain injection of rAAV and no signs of inflammatory reaction were detected by immunostaining for the glial fibrillary acidic protein (GFAP) or the activated microglial marker, iba1 (Supplementary Figure S3). In some experiments, animals were first analysed to confirm a coincident and comparable expression of the EGFP mRNA and miR-34a transcript by Real-Time PCR on cortex and hippocampus (Supplementary Figure S4 a).

Using EGFP native fluorescence to directly detect transgene expression, we found a diffuse transduction of different brain regions also distant from the site of injection. Cells in the hippocampus, cortex, striatum, olfactory bulb, cerebellum, amygdala, hypothalamus were extensively transduced, confirming rAAV as a powerful tool to target brain cells for genetic manipulation (Supplementary Figure S4 b and data not shown). In the neocortex, we found EGFP-positive cells scattered in all layers, more numerous in layer II-III (Supplementary Figure S4 b). Since native EGFP fluorescence might underestimate transduction efficiency, we co-labelled brain sections with an anti-GFP antibody. Co-labelling experiments showed efficient transduction of the hippocampus, with abundant co-labelled cells in the DG (Supplementary Figure S4 c).

It is noteworthy that, despite the lack of specificity of CMV and PGK promoters, the rAAV-mediated overexpression resulted neuron-specific and we never found infected glial cells (Supplementary Figure S4 d), as revealed by double labelling using antibodies against the neuronal marker NeuN and the glial marker GFAP. This observation is consistent with the previously reported tropism of rAAV toward neurons.[4, 5]

Supplementary Materials and Methods

Expression Constructs

The vectors used to construct and package rAAVs have been previously described,[6, 7] and were kindly provided by Hilmar Bading, University of Heidelberg, Germany.[8]

Briefly, the pri-miR-34a was cloned by PCR (Finzyme) (gene ENSRNOG00000035623, from position 291 to 1124), using specific primers (MWG-Biotech, Germany) from rat genomic DNA and inserted into the XhoI and EcoV sites of Tween vector.[9, 10] Later, the region containing the CMV promoter driving the pri-miR-34a, the PGK promoter driving the enhanced GFP (EGFP) from the Tween vector was subclone into an AAV plasmid backbone, containing the woodchuck post-transcriptional regulatory element (WPRE) and the bovine growth hormone polyA (bGH), to yield the construct pAAV-miR-34a. The same pAAV-CMV-WPRE-bGH backbone carrying no miR (pAAV-empty vector), but expressing the only EGFP, was used as controls. It was obtained removing the pri-miR-34a by cutting with XbaI, blunting by Klenow polymerase and ligating with the EcoRV site of the AAV backbone. Cloning orientation was verified by diagnostic cuts followed by sequencing.

Three web-based miRNA target prediction methods were used: miRanda (http://www.microrna.org), TargetScan (http://www.targetscan.org) and PicTar (http://pictar.mdc-berlin.de). The 3' UTR of DCX (sequence ENSRNOT00000074322, NM_053379.3 segment from 1702 position to 2717), including the predicted sequence target of miR-34a, were amplified by PCR from rat brain cDNA using specific primers (see Table S1, Supplementary information), and subsequently, cloned into XhoI-NotI sites of the psiCheck2 vector (Promega). Moreover, to ablate the miR-34a binding site in the 3' UTR of DCX inserted into psiCheck2 vector, and specifically modify the nucleotides in the seed

sequence, we designed specific primers (see Table S1, Supplementary information) according to the QuikChange® Site-Directed Mutagenesis Kit (Stratagene). The amplified mutated 3' UTR DCX fragment included a new BamHI restriction site to facilitate diagnostics.

Real-Time PCR

Total RNA from neuronal cells or dissected brain regions was isolated using Trizol (Invitrogen) according to the manufacturer's instructions. RNA samples were treated with RNase-free DNase I (Invitrogen). Total RNA was reverse transcribed using Superscript III reverse transcriptase and oligo(dT) and random examer primers (Invitrogen). Relative quantitative real-time PCR was performed in a Real-Time Thermocycler (MX 3000, Stratagene) with SYBR green ready mix (Stratagene) and specific primers (see Table S1, Supplementary information). The primers to amplify the miR-34a were designed at the stem region of the precursor form as previously described.[11] Similar amplification results were obtained using primers and conditions from the miRcury LNA PCR system (Exigon). The expression of miR-34a and each other gene was defined, from the threshold cycle (C_t), and relative expression levels were calculated by using the $2^{-\Delta\Delta C_t}$ method after normalization with reference to expression of 18S or 5S rRna and U6 small RNA (for miR-34a quantitation in exosome preparation).

Supplementary Figures

Title:

miR-34a regulates cell proliferation, morphology and function of newborn neurons resulting in improved behavioural outcomes

Cristiana Mollinari 1-2, Mauro Racaniello 2, Alessandra Berry 2, Massimo Pieri 3-4, Maria Chiara de Stefano 2, Alessio Cardinale 5 Cristina Zona 4, Francesca Cirulli 2, Enrico Garaci 5*, Daniela Merlo 2*

*** Corresponding Authors**

Supplementary Figures S1-S4

Mollinari et al., Figure S1

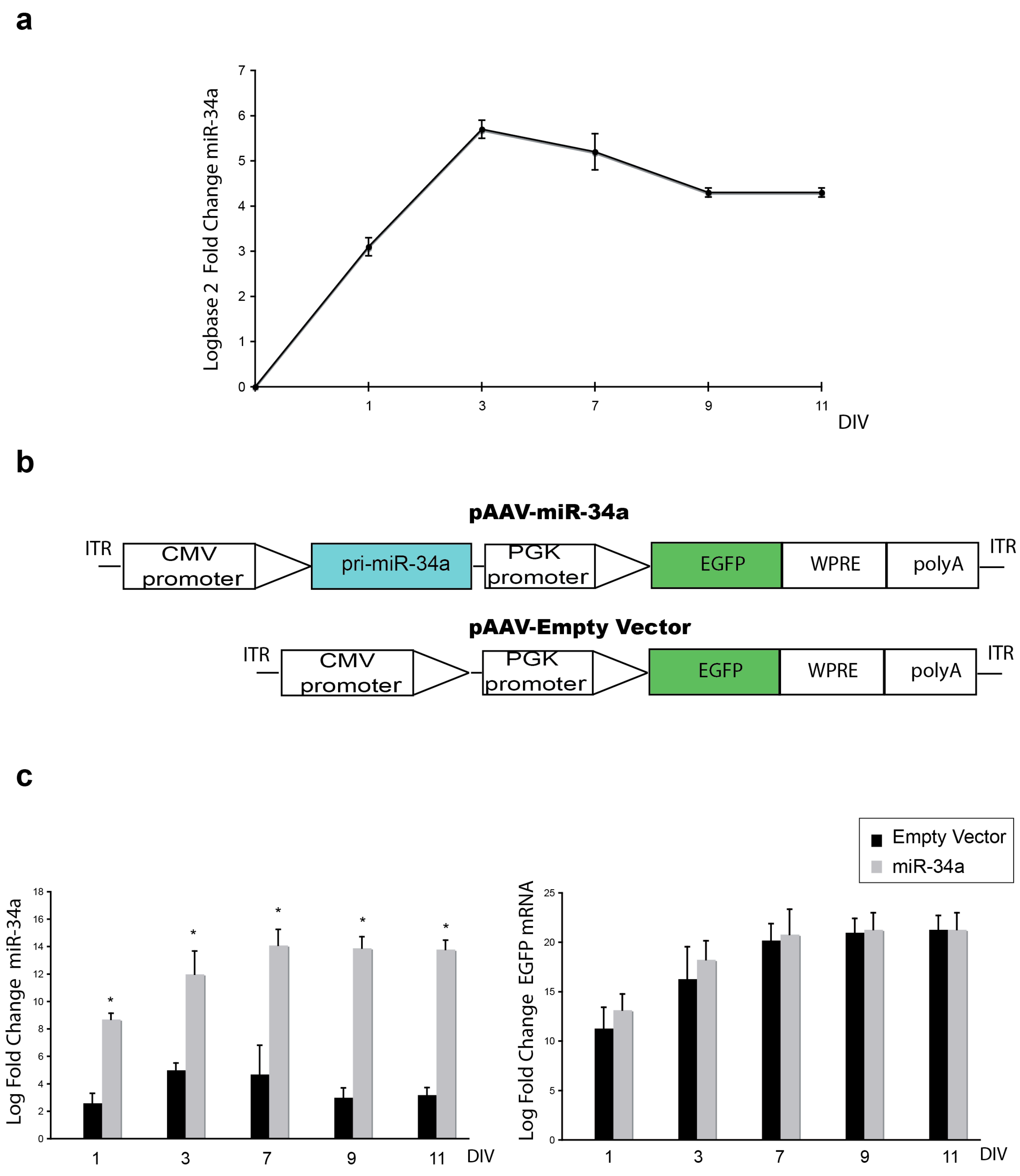

Supplementary Figure S1

miR-34a expression levels are modulated during differentiation of rat embryonic

cortical precursors

(**a**) Real-time PCR of miR-34a was performed on total RNA from developing rat cortical precursors at different DIV. A significant up regulation of the endogenous miR-34a expression was found from the early stages of differentiation remaining high and stable in the tardive stages. Values are plotted as log (base2) fold change to calibrator (undifferentiated cells at DIV 0). 18S rRNA and 5S rRNA expression were used for each sample normalization.

(**b**) rAAV vector scheme: pAAV-miR-34a construct used to express the pri-miR-34a under the control of the constitutive cytomegalovirus (CMV) promoter, along with the EGFP under the control of the constitutive phosphoglycerate kinase (PGK) promoter. pAAV-Empty Vector expresses only EGFP and represents the control vector. Inverted terminal repeats (ITR); woodchuck post-transcriptional regulatory element (WPRE) and the bovine growth hormone polyA (bGH) are contained within the rAAV vectors.

(**c**) Real-time PCR analysis performed on total RNA from cortical cultures maintained for different DIV. Please, note the high level of expression mediated by transduction with the pAAV-miR-34a for both the miR-34a (left histogram) and the EGFP (right histogram) transcripts. Differently, transduction with the pAAV-Empty Vector is accompanied exclusively by the overexpression of EGFP transcript (right histogram). Expression is plotted as log (base2) fold change relative to levels in cells at DIV 0. Data were normalized to the expression of 18S rRNA and 5S rRNA. *P<0,01 compared with control values (undifferentiated cells DIV 0).

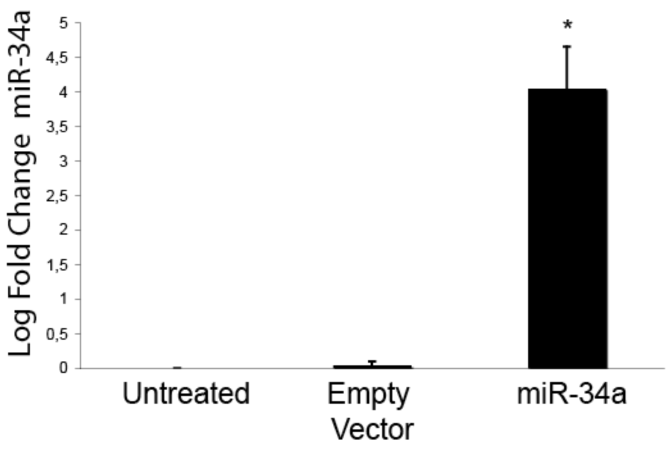

Supplementary Figure S2

miR-34a expression levels in exosomes isolated from culture medium of rat embryonic cortical neurons

Real-time PCR for miR-34a transcript was performed on RNA isolated from exosomes of cortical neuronal cultures (DIV 7). Values are plotted as log (base2) fold change to calibrator (uninfected cultures). 5S rRNA and U6 were used for each sample normalization. *P<0,05 compared with control values.

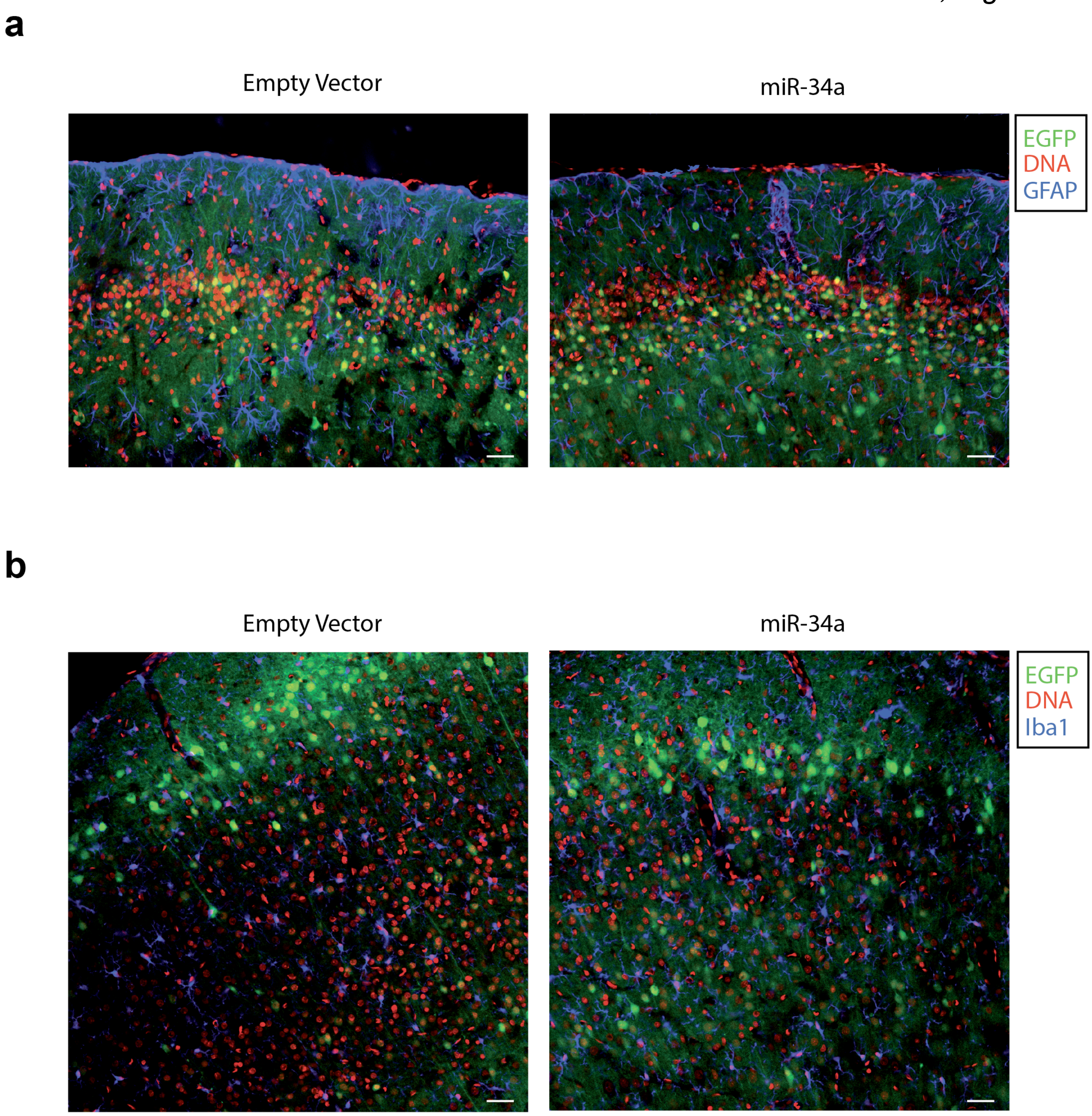

Supplementary Figure S3

rAAV infection is highly efficient in miR delivery after ventricular injection of rat pups with no sign of inflammation and tissue damage.

(**a**) Confocal images positive for direct EGFP fluorescence immunostained for GFAP (blue) and counterstained with DAPI for detection of nuclei (red).

(**b**) Confocal images positive for direct EGFP fluorescence immunostained for iba1 (blue) and counterstained with DAPI (red). rAAV ventricular injections did not affect normal architectural structure of cortical brain tissues and did not induce inflammatory responses as revealed by the staining with the anti-GFAP (glial cells) and anti-Iba1 (microglial cells). Scale bar: 10 μm.

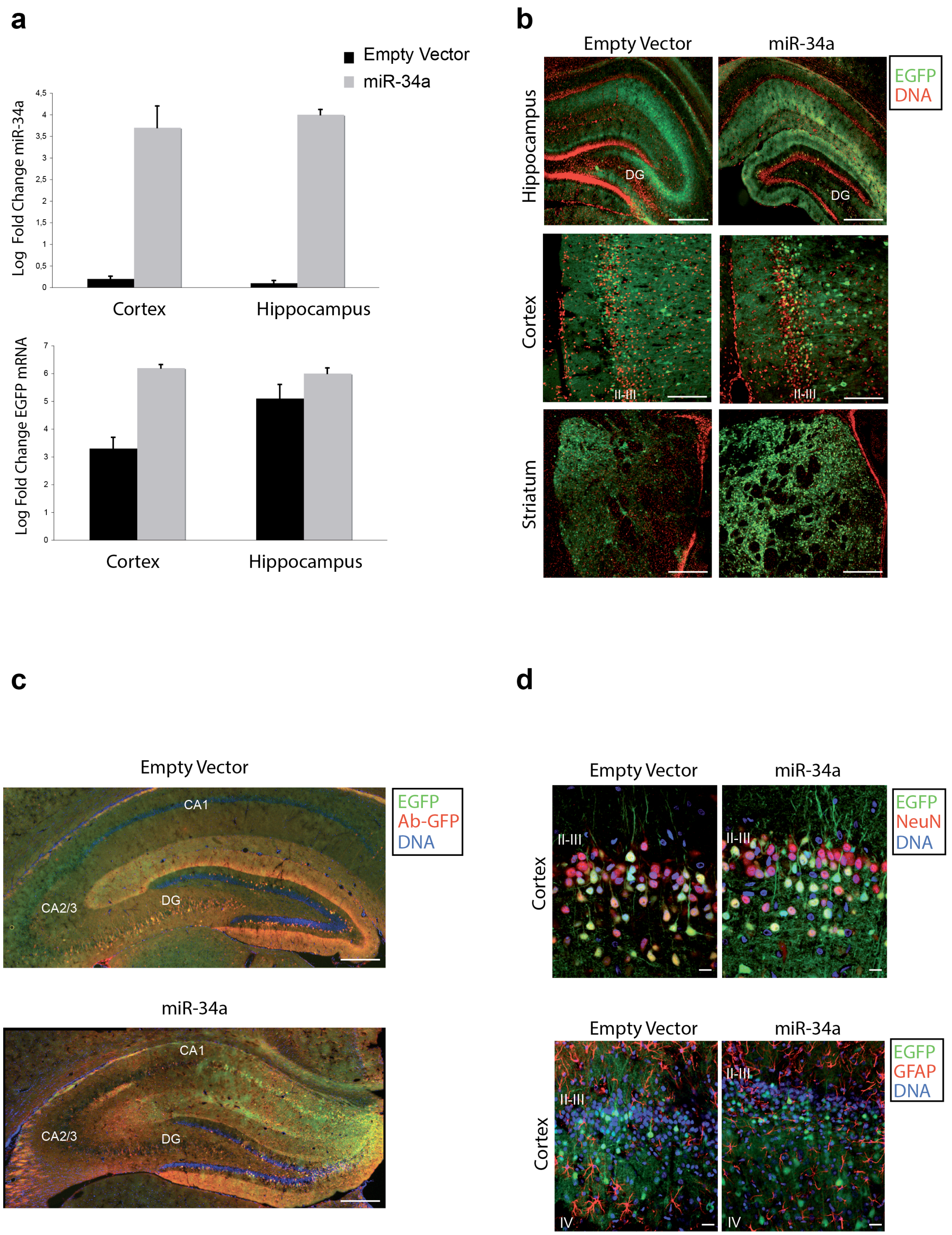
a
Empty Vector
miR-34a
Log Fold Change miR-34a
4,5
4
3,5
3
2,5
2
1,5
1
0,5
0
Cortex
Hippocampus
Log Fold Change EGFP mRNA
7
6
5
4
3
2
1
0
Cortex
Hippocampus
b
Empty Vector
miR-34a
EGFP
DNA
Hippocampus
DG
DG
Cortex
II-III
II-III
Striatum
c
Empty Vector
EGFP
Ab-GFP
DNA
CA1
CA2/3
DG
miR-34a
CA1
CA2/3
DG
d
Empty Vector
miR-34a
EGFP
NeuN
DNA
Cortex
II-III
II-III
Empty Vector
miR-34a
EGFP
GFAP
DNA
Cortex
II-III
II-III
IV
IV

Supplementary Figure S4

miR-34a is highly and widely expressed after single neonatal ventricular injection of rAAV and exclusively affects neuronal cells of rat brain.

(**a**) (Left panel) Real-Time PCR analysis of miR-34a (upper histogram) and EGFP transcripts (lower histogram) of RNA isolated from cortex and hippocampus of injected animals after five weeks from virus inoculation. The histograms show a high expression of miR-34a along with the EGFP in both brain regions of rAAV-miR-34a injected animals. Differently, only EGFP transcript overexpression is evident in both brain regions of rAAV-Empty Vector injected animals. Values are mean ± s.e.m., as compared with control values of uninfected brain regions.

(**b**) Direct EGFP fluorescence (green) of different brain regions counterstained with DAPI for detection of nuclei (red), shows robust gene delivery in hippocampus, cortex and striatum. DG: dentate gyrus; II-III: neocortical layers. Note the relatively normal structures of the infected brain regions after rAAV injection. Scale bar: 100 μm.

(**c**) Confocal images positive for EGFP fluorescence and stained with an anti-GFP antibody (red) and DAPI (blue). The anti-EGFP staining allows an even better identification of cells infected by the rAAV vectors and confirms a robust gene delivery in the hippocampus and in granule cells of the DG. Scale bar: 100 μm.

(**d**) (Upper panel). Confocal images of infected cells in the neocortex (layer II-III) positive for EGFP and labelled for NeuN (red) and DAPI (blue). The co-localization of EGFP fluorescence with NeuN, confirms the specificity of rAAV–mediated infection for neurons. (Lower panel). Confocal images of infected cells in the neocortex (layer II-III) positive for EGFP and immunostained for GFAP (red) and counterstained with DAPI (blue). No glial cells appear infected by the rAAV vectors. Scale bar: 5 μm.

miR-34a (rAAV) for	5' CCGCTGAGTCCTCTGTAAGAGCAGCA 3'
miR-34a (rAAV) rev	5' TCCGATATCAGCGTTGCATGGAGCA 3'
Rat miR-34a for	5' CGGTGCTGAGTAATTCTTTGG 3'
Rat miR-34a rev	5' TGCAGCACTTCTAGGGCAGTA 3'
18S for	5' GTAACCCGTTGAACCCCATT 3'
18S rev	5' CCATCCAATCGGTAGTAGCG 3'
5S for	5' CCATACCACCCTGAACGTG 3'
5S rev	5' CCTACAGCACCCGGTATCC 3'
U6 for	5' GCTTCGGCAGCACATATACTAA 3'
U6 rev	5' AAAATATGGAACGCTTCACG 3'
NUMBL for	5' GGTGGATGACAAGACCAAGG 3'
NUMBL rev	5' GTGCCATCACGGCATATGTA 3'
NOTCH1 for	5' CTCACGCTGATGTCAATGCT 3'
NOTCH1 rev	5' CTTTGTTGGCTCCGTTCTTC 3'
DCX for	5' AAAAGCTTCCCCAACACCTC 3'
DCX rev	5' AAGTTCCATTTGCGTCTTGG 3'
3'UTR DCX for	5' CCGCTCGAGGAGAAACACCAGCGACTTCC 3'
3'UTR DCX rev	5' ATTTGCGGCCGCAAACGACATGTTGGCTTTGA 3'
MUT3'UTR DCX for	5'GCAGAAGAATACTGTAGTTTAGAAATCATGCTCTTTA ATGGATCCAGAAAGCTCCGGG 3'
MUT3'UTR DCX rev	5'CCCGGAGCTTTCTGGATCCATTAAAGAGCATGATTT CTAAACTACAGTATTCTTCTGC 3'
EGFP for	5' TGCACCATCGACGTCTACAT 3'
EGFP rev	5' GTCTCTGGCCATTTTGGAGA 3'
WPRE for	5' ACTGTGTTTGCTGACGCAAC 3'
WPRE rev	5' CAACACCACGGAATTGTCAG 3'

Supplementary Table S1

Primers used in this study

miR-34a, microRNA-34a; 18S, 18 S ribosomal RNA; 5S, 5S ribosomal RNA; U6, U6 small nuclear RNA; NUMBL, Numb-like protein; Notch1, Notch Homolog 1 Translocation-associated (Drosophila); DCX, Doublecortin; 3'UTR, 3'Untraslated Region; MUT 3'UTR DCX, Mutated 3'Untraslated Region Doublecortin; EGFP, Enhanced Green Fluorescent Protein; WPRE, Woodchuck Post-transcriptional Regulatory Element.

Supplementary References

1. Snyder RO. Adeno-associated virus-mediated gene delivery. *J Gene Med* 1999, **1**(3)**:** 166-175.

2. During MJ, Samulski RJ, Elsworth JD, Kaplitt MG, Leone P, Xiao X*, et al.* In vivo expression of therapeutic human genes for dopamine production in the caudates of MPTP-treated monkeys using an AAV vector. *Gene Ther* 1998, **5**(6)**:** 820-827.

3. Rivera VM, Ye X, Courage NL, Sachar J, Cerasoli F, Jr., Wilson JM*, et al.* Long-term regulated expression of growth hormone in mice after intramuscular gene transfer. *Proc Natl Acad Sci U S A* 1999, **96**(15)**:** 8657-8662.

4. Bartlett JS, Samulski RJ, McCown TJ. Selective and rapid uptake of adeno-associated virus type 2 in brain. *Hum Gene Ther* 1998, **9**(8)**:** 1181-1186.

5. Burger C, Nash K, Mandel RJ. Recombinant adeno-associated viral vectors in the nervous system. *Hum Gene Ther* 2005, **16**(7)**:** 781-791.

6. Zhang C, Cortez NG, Berns KI. Characterization of a bipartite recombinant adeno-associated viral vector for site-specific integration. *Hum Gene Ther* 2007, **18**(9)**:** 787-797.

7. Zhang H, Xie J, Xie Q, Wilson JM, Gao G. Adenovirus-adeno-associated virus hybrid for large-scale recombinant adeno-associated virus production. *Hum Gene Ther* 2009, **20**(9)**:** 922-929.

8. Lau D, Bading H. Synaptic activity-mediated suppression of p53 and induction of nuclear calcium-regulated neuroprotective genes promote survival through inhibition of mitochondrial permeability transition. *J Neurosci* 2009, **29**(14)**:** 4420-4429.

9. Ricci-Vitiani L, Pedini F, Mollinari C, Condorelli G, Bonci D, Bez A*, et al.* Absence of caspase 8 and high expression of PED protect primitive neural cells from cell death. *J Exp Med* 2004, **200**(10)**:** 1257-1266.

10. Mollinari C, Ricci-Vitiani L, Pieri M, Lucantoni C, Rinaldi AM, Racaniello M*, et al.* Downregulation of thymosin beta4 in neural progenitor grafts promotes spinal cord regeneration. *J Cell Sci* 2009, **122**(Pt 22)**:** 4195-4207.

11. Dutta KK, Zhong Y, Liu YT, Yamada T, Akatsuka S, Hu Q*, et al.* Association of microRNA-34a overexpression with proliferation is cell type-dependent. *Cancer Sci* 2007, **98**(12)**:** 1845-1852.
